# Einsatz neuer Medien in der pädiatrischen Psychosomatik

**DOI:** 10.1007/s00112-021-01184-y

**Published:** 2021-04-15

**Authors:** A. Felnhofer, L. Fischer-Grote

**Affiliations:** 1 Univ.-Klinik für Kinder- und Jugendheilkunde, Pädiatrische Psychosomatik, Medizinische Universität Wien, Währinger Gürtel 8–20, Fach 27, 1090 Wien, Österreich; 2 Comprehensive Center for Pediatrics, CCP, Medizinische Universität Wien, Wien, Österreich

**Keywords:** Smartphone, Virtuelle Realität, Computerspiele, Chronische Erkrankung, Psychische Störungen, Smartphone, Virtual reality, Computer games, Chronic disease, Mental disorders

## Abstract

Neue Medien wie Smartphone-Apps oder virtuelle Realitäten (VR) finden zunehmend Anwendung in der pädiatrischen Psychosomatik. In der Diagnostik liegen die Vorteile in der Erfassung von Daten im Alltag sowie in der realitätsnahen und zugleich standardisierten Erfassung mithilfe der VR. In der Behandlung lassen sich selbstadministrierte und hybride Technologien von computerassistierten und computerspielbasierten Interventionen unterscheiden, die allesamt zunehmend in der pädiatrischen Psychosomatik zum Einsatz kommen, so z. B. bei Schmerzerkrankungen, Enkopresis, chronischen Erkrankungen oder auch begleitenden Depressionen und Ängsten. Durch das Auslösen alltagsnaher Reaktionen bei gleichzeitiger maximaler Kontrolle bieten VR auch in der Forschung große Vorteile. Nichtsdestotrotz sind Kontraindikationen wie Psychosen, Epilepsie und Migräne zu beachten. Eine umfassende Schulung des Fachpersonals ist daher für die Nutzung neuer Medien in der Diagnostik, Behandlung und Forschung essenziell.

## Abstract (en)

New media such as smartphone apps and virtual reality (VR) are increasingly being used in pediatric psychosomatic care. The advantages concerning diagnostic assessments lie in the collection of data in daily life as well as in a realistic and standardized data collection with VR. With respect to treatment, self-administered and hybrid technologies can be distinguished from computer-assisted and computer gaming-based interventions. They are all applied in pediatric psychosomatic care to an increasing extent, e.g. in the treatment of pain, encopresis, chronic illnesses as well as comorbid depressive and anxiety disorders. The utilization of VR also offers great advantages in research due to eliciting true to life reactions, while simultaneously providing maximum control. Nevertheless, contraindications, such as psychosis, epilepsy and migraine must be considered. An extensive training of professionals is therefore necessary for the application of new media in diagnostics, treatment and research.

Neue Medien definieren nicht nur den Alltag heutzutage aufwachsender Kinder und Jugendlicher, sondern halten – begünstigt durch die „Coronavirus-disease-2019“(COVID-19)-Krise – auch vermehrt Einzug in die Pädiatrie. Die Anwendungsmöglichkeiten reichen von der Diagnostik über die Therapie bis hin zur Forschung. Der vorliegende Beitrag setzt sich zum Ziel, einen umfassenden, wenn auch nicht erschöpfenden Überblick über den Einsatz neuer Medien in der pädiatrischen Psychosomatik zu bieten und so einen Grundstein für deren gewinnbringende Nutzung zu legen.

## Neue Medien – eine Eingrenzung

Wenngleich der Begriff „neue Medien“ aufgrund seiner breiten Definition ebenso schwer zu fassen ist wie die Vielfalt an Technologien, die er bezeichnet, sollen hiermit für den vorliegenden Beitrag v. a. jene interaktiven Technologien gemeint sein, die sich in den letzten Jahrzehnten zusehends weiterentwickelt haben und nun unseren Alltag prägen [14]: das Internet und seine Dienste (z. B. soziale Netzwerke, Games, Apps) sowie auch virtuelle Realitäten (VR). Mit der fortschreitenden Entwicklung dieser Medien steigt auch deren Anwendungsvielfalt, darunter der Einsatz bei Kindern und Jugendlichen.

Hier ist der Vorteil gegenüber herkömmlichen Behandlungsmethoden besonders evident: Während klassische Therapien häufig vor der Herausforderung stehen, die Motivation und Adhärenz möglichst aufrechtzuerhalten, gelingt dies technologiebasierten Behandlungsmethoden – bei einer Kombination von spielerischen Elementen (z. B. Narrativ, Incentives) gemäß dem Gamification-Ansatz [25] häufiger. Erfolgreiche Anwendungsgebiete umfassen u. a. den Einsatz von VR bei Kindern mit Zerebralparese [23] und Autismus-Spektrum-Störungen (ASS, [19]) oder auch in der pädiatrischen Rehabilitation [22]. Ebenso ist ein Vorteil für all jene Kinder und Jugendlichen zu erwarten, die an einer pädiatrisch psychosomatischen Abteilung – z. B. mit somatoformen Schmerzstörungen, Ausscheidungsstörungen (Enuresis, Enkopresis) oder einer chronischen Erkrankung und komorbiden psychischen Störung – vorstellig werden. Nachfolgend sollen daher jene Ansätze aufbereitet werden, die der Diagnostik, Behandlung und Forschung mit pädiatrisch-psychosomatischen PatientInnen gewidmet sind.

## Diagnostik

In zunehmendem Umfang kommen in der psychosomatischen Diagnostik Methoden zum Einsatz, die neue Medien nutzen. Ein Beispiel ist die Anwendung von Echtzeitalltagsmessungen, das „ecological momentary assessment“ (EMA), das es ermöglicht, wie mit einem elektronischen Tagebuch alltagsnah Informationen zu bestimmten Zeitpunkten oder nach bestimmten Ereignissen zu sammeln. Das Assessment erfolgt u. a. über Smartphones via Apps, Textnachrichten oder webbasierte Fragebogen [13, 30]. So können z. B. Kinder und Jugendliche mit Migräne mehrmals täglich bei gleichzeitiger Erfassung von Umgebungsvariablen wie dem Wetter befragt werden [4], oder Jugendliche mit Diabetes [12] hinsichtlich ihrer sozialen Interaktionen, Stimmungslage und Blutglucosemessung.

Ein weiteres Beispiel stellt der Gebrauch von VR dar. Der Vorteil liegt in einer möglichst lebensechten und zugleich standardisierten Erfassung von Symptomen [29]. So kann z. B. ein virtuelles Klassenzimmer [10] zur Erfassung von Exekutivfunktionen bei Neurofibromatose Typ 1 dienen. Dabei wird ein Klassenzimmer simuliert, in dem auf bestimmte Reize, die auf der virtuellen Tafel erscheinen, reagiert werden soll, während die Reaktion auf andere Reize inhibiert wird. Besondere Realitätsnähe wird durch den Einsatz von auditiven, visuellen oder visuell-auditiven Störreizen erzeugt. Ebenso kann z. B. die Emotionserkennung von Jugendlichen mit ASS mithilfe von virtuellen Avataren, die verschiedene Gesichtsausdrücke zeigen, diagnostiziert werden [1].

## Behandlung

Auch in der klinisch-psychologischen bzw. psychotherapeutischen Behandlung nimmt der Einsatz neuer Medien zu. Dabei lassen sich, ausgehend vom Ausmaß des therapeutischen Kontakts und dem Einsatzgebiet, 4 Gruppen von Technologien unterscheiden ([6, 28]; Tab. 1).

**Table 1:** 

Art der Technologie	Einsatzbereiche	Beispiele für Inhalte	Existierende Programme
Selbstadministrierte Technologien	Selbstständig administrierbar; *Nachsorge* (Rehabilitation, Rückfallprävention)	Internetbasierte Psychoedukation	U‑CAN-POOP-TOO [24]
Hybride Technologien	Persönlicher Kontakt und selbstständige Administration; *ambulante Versorgung* (Patientenmanagement, Früherkennung)	Verhaltensaufzeichnung und Aufzeichnung von Krankheitsdaten über *Apps*	Young.constant-care.com (YCC, [3])
		Vermittlung von* KVT-basierten Interventionen*	Web-based Management of Adolescent pain (Web-MAP2, [20])
Computerassistierte Interventionen	Durchführung mit BehandlerIn; *KVT-basierte Interventionen*	*Virtual Reality Exposure Therapy (VRET)*	Conquer Catharsis [17]
		Entspannung mit VR	VR bei Schulangst [11]
Computerspielbasierte Interventionen	Spielerische Elemente werden mit therapeutischen Elementen kombiniert; *Behandlung/Therapie*	*Serious Games*	SPARX [18, 9]
			MindLight [27]
			KidBreath [5]

*KVT* kognitiv-verhaltenstherapeutisch, *VR* virtuelle Realität

Die erste Gruppe stellen selbstadministrierte Technologien dar, die v. a. im Kontext der Rehabilitation und Rückfallprophylaxe eingesetzt werden [6, 28]. So kommen z. B. bei der internetbasierte Intervention *U‑CAN-POOP-TOO* für Kinder mit Enkopresis [24] verschiedene psychoedukative Module zum Einsatz, die z. B. hinsichtlich anatomischer Grundlagen sowie medikamentöser und verhaltensbezogener Behandlungsaspekte informieren.

Der Gamification-Ansatz erleichtert die Aufrechterhaltung von Motivation und Adhärenz der Patienten

Im Gegensatz zu rein selbstadministrierten Technologien werden bei hybriden Technologien die autonom angewandten Module durch einen direkten Kontakt ergänzt. Diese Kombination eignet sich besonders für die ambulante Versorgung. Kritische Situationen können rechtzeitig erkannt und erlernte Strategien im Alltag ausprobiert werden [28]. Beispielsweise können Kinder und Jugendliche mit chronisch entzündlichen Darmerkrankungen monatlich Daten zur Erkrankung online eingeben und diese durch Blut- und Stuhlproben ergänzen. Basierend auf den resultierenden Werten werden nach einem Ampelsystem Empfehlungen gegeben bzw. die PatientInnen zu einem persönlichen Termin eingeladen [3]. Ähnlich ermöglicht eine internetbasierte kognitiv-verhaltenstherapeutische Intervention es Kindern und Jugendlichen mit chronischen Schmerzen, diverse therapeutische Module (z. B. zu Entspannungstechniken, kognitiven Bewältigungsstrategien) selbst zu durchlaufen, während sie von einem Online-Coach unterstützt werden [20].

Hybride Technologien eignen sich besonders für die ambulante Versorgung

**Figure 1:**
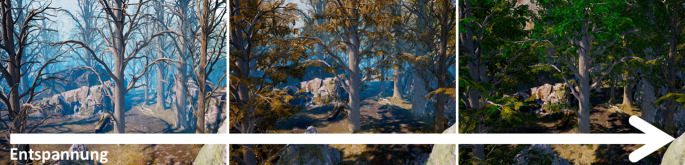

Die dritte Gruppe stellen computerassistierte Interventionen dar, deren Anwendung in Anwesenheit der BehandlerInnen erfolgt. Neben Computertrainings gehören auch VR zu dieser Gruppe [6]. So wird beispielsweise in der Anwendung *Conquer Catharsis* [17] Biofeedback mit VR kombiniert, indem Änderungen der Herzrate (HR) mit Veränderungen in der virtuellen Computerumgebung einhergehen (z. B. ergrünen Bäume, je weiter die HR sinkt; Abb. 1). Derzeit wird das Programm bei Kindern und Jugendlichen mit stressbedingten Störungen (z. B. Depressionen, Angst) evaluiert. Ziel des Trainings ist, Entspannungstechniken zu erlernen und eine verbesserte Selbstwirksamkeit zu erreichen. Auch in der Behandlung von Schulangst kann VR eingesetzt werden, indem die Schulumgebung und der Klassenraum realitätsnah simuliert werden [11].

Als vierte Gruppe lassen sich spielbasierte Interventionen beschreiben, die als „serious games“ therapeutische Elemente in Computerspiele integrieren [28]. Anwendung finden Serious games beispielsweise in der Behandlung von Depressionen, Angststörungen oder chronischen somatischen Erkrankungen. Das Serious game *SPARX* („*s*mart, *p*ositive, *a*ctive, *r*ealistic, X‑factor thoughts“, [9, 18]) ist ein dreidimensionales Fantasy-Rollenspiel, in dem Jugendliche mit depressiven Störungen Avatare, die personifizierte negative automatische Gedanken verkörpern, in aufsteigenden Levels bekämpfen müssen. Dabei kommen therapeutische Inhalte wie kognitive Umstrukturierung, Achtsamkeit und Skills zum Einsatz. *MindLight* [26, 27] hingegen ist ein Serious game im Adventure-Stil, das für die Behandlung von Angststörungen eingesetzt wird. Die SpielerInnen lernen, ihre Ängste mithilfe von Neurofeedback, Exposition und Aufmerksamkeitsbiasmodifikation zu kontrollieren. Ebenso können auf der E‑Learning Plattform *KidBreath *Kinder und Jugendliche mit Asthma auf spielerische Art den Umgang mit ihrer Erkrankung verbessern [5].

## Forschung

Eine zentrale Herausforderung in der Erforschung diverser Erkrankungen und assoziierter bzw. bedingender Phänomene (z. B. Stress, Angst) liegt in der Balance zwischen einer möglichst großen experimentellen Kontrolle (d. h. dem Ausschalten externer Störeinflüsse) und der Sicherstellung der ökologischen Validität, d. h., dass die unter Laborbedingungen beobachteten Reaktionen mit Alltagsreaktionen vergleichbar sind [21]. So werden z. B. bei der Erforschung sozialer Phänomene meist SchauspielerInnen eingesetzt, die jedoch nicht immer exakt gleich reagieren können und somit eine potenzielle Fehlerquelle darstellen [15]. Ebenso hat die Erfassung von Fähigkeiten unter Laborbedingungen (z. B. Computertests für Aufmerksamkeitsfunktionen) den Nachteil, dass die Leistungen möglicherweise aufgrund der mangelnden Komplexität der Situation verfälscht werden.

In der Forschung ermöglichen VR die nötige Kontrolle über die Zielbedingungen

Als Lösung für dieses scheinbar unauflösliche Dilemma haben sich VR zusehends bewährt: Diese bieten nicht nur den sensorischen Reichtum von Alltagsumgebungen, sondern ermöglichen es, die nötige Kontrolle über die Zielbedingungen auszuüben. Entsprechend konnten Studien aufzeigen, dass die Reaktionen in VR mit jenen unter Realbedingungen übereinstimmen und sich somit VR für das Studium menschlichen Erlebens und Verhaltens hervorragend eignet [2].

So können Avatare eingesetzt werden, um eine Vielfalt sozialer Situationen nachzustellen und die Auswirkungen u. a. von Mobbing in Form von sozialem Ausschluss auf die Stressverarbeitung [16] oder auch von sozialer Unterstützung als Resilienzfaktor [8] zu untersuchen. Ebenso können verschiedene Fähigkeiten wie z. B. Aufmerksamkeitsprozesse, kognitive Leistungen bzw. angstbesetzte Situationen (z. B. ein Schulreferat, schmerzhafte medizinische Prozeduren) durch deren kontextuelle Einbettung realitätsnah im Labor getestet und so bessere Vorhersagen über entsprechende Beeinträchtigungen getroffen werden [21].

## Herausforderungen

Seitens der Fachkräfte erfordert der Einsatz neuer Medien eine entsprechende Vorbereitung und ein grundlegendes Know-how [7]. Die letzten Jahrzehnte haben eine Vielzahl leistbarer Technologien hervorgebracht, die jedoch nicht alle für den medizinischen Gebrauch geeignet sind. Zunächst bedarf es einer eingehenden Prüfung der jeweiligen Applikation, ob diese den Einsatzkriterien standhält und der Zielerreichung dienlich ist. Während einige Technologien selbstadministriert angewendet werden können, ist bei VR – insbesondere bei jenen, die eine VR-Brille verwenden – dringend davon abzuraten. Im Bereich der Therapie kann VR nur auf Basis eines evidenzbasierten Behandlungskonzeptes eingesetzt werden, das – nicht zuletzt aufgrund des Potenzials von VR, intensive emotionale Reaktionen hervorzurufen – eine Vor- und Nachbesprechung durch qualifiziertes Personal erfordert [7].

Zusätzliche Herausforderungen umfassen die eingeschränkte Verwendbarkeit von VR bei Vorliegen einer Migräne, Epilepsie oder akut psychotischen Zuständen. Ebenso ist bei Personen mit bekannter Kinetose (Reisekrankheit) Vorsicht geboten, da diese vermehrt mit einer sog. Cybersickness, d. h. Schwindel oder Übelkeit, auf die VR reagieren können. Ferner sind kommerzielle VR-Brillen für Erwachsene konzipiert, sodass sie für sehr junge Kinder (je nach Körpergröße < 8 Jahren) meist ungeeignet sind. Zuletzt gilt es bei der therapeutischen Anwendung von internetbasierten Technologien oder Apps zu bedenken, dass die Anforderungen an einen sicheren und eigenverantwortlichen Einsatz aufgrund diverser Umstände (z. B. Fehlen der technischen Voraussetzungen wie Internetzugang, eigenes Smartphone) nicht gegeben sein und somit einem Behandlungserfolg im Wege stehen können [14].

## Konklusion und Ausblick

Angesichts der vielgestalten Möglichkeiten, die neue Medien bieten, ist es wünschenswert, dass diese Technologien in Zukunft Einzug in die Arbeit mit pädiatrischen PatientInnen halten. So wären hinsichtlich der Steigerung der Adhärenz speziell bei therapieresistenten Kindern und Jugendlichen eine Integration in den Behandlungsalltag sowie auch die Aufrüstung der ambulanten Versorgung unter Einbezug hybrider Formen gewinnbringend. Gleichsam ist zu erwarten, dass VR-basierte Forschung bis dato ungekannte Erkenntnisse über die Entstehung und Aufrechterhaltung psychosomatischer Erkrankungen nach sich ziehen wird. Grundlage für einen gelungenen Einsatz – ob in der Diagnostik, Behandlung oder Forschung – stellen jedoch eine entsprechende wissenschaftlich fundierte Ausbildung sowie auch Schulungen und kontinuierliche, den jeweiligen Entwicklungen angepasste Fortbildungen von Fachkräften dar.

## Fazit für die Praxis

- Ecological momentary assessments (EMA) ermöglichen die diagnostische Informationserhebung im Alltag.
- Virtuelle Realitäten (VR) eignen sich für die Diagnostik aufgrund der besonders realitätsnahen Erhebung von Symptomen.
- Selbstadministrierte und hybride Technologien sowie computerassistierte und computerspielbasierte Interventionen bieten neue Behandlungsmöglichkeiten im Kontext der pädiatrischen Psychosomatik.
- Da VR nicht nur alltagsnahe Reaktionen auslösen können, sondern auch eine maximale Kontrolle über die Bedingungen ermöglichen, eignen sie sich hervorragend für die Forschung.
- Keinesfalls sollten VR im Rahmen der Behandlung selbstadministriert angewendet werden, sondern der Einsatz sollte stets einem evidenzbasierten Behandlungskonzept folgen.
- Zu den Kontraindikationen von VR zählen Migräne, Epilepsie und akute Psychosen.
- Zentral für einen erfolgreichen Einsatz neuer Medien ist eine entsprechende Schulung der Fachkräfte.
